# A refractory head tremor appearing after volatile anesthesia combined with epidural anesthesia in a patient with spinocerebellar ataxia type 6

**DOI:** 10.1186/s40981-018-0150-9

**Published:** 2018-01-30

**Authors:** Takaya Nishida, Masayori Nakajima

**Affiliations:** Department of Anesthesiology, Takatsuki General Hospital, 1-3-13, Kosobe-cho, Takatsuki-shi, Osaka-fu 569-1192 Japan

**Keywords:** Spinocerebellar ataxia type 6, Volatile anesthesia, Surgery, Extrapyramidal symptoms

## Abstract

A 64-year-old female patient with spinocerebellar ataxia type 6 was referred to our department for pancreatic cancer and anesthetized with volatile anesthesia combined with epidural anesthesia for pancreaticoduodenectomy. No complications arose during surgery. On postoperative day 4, a head tremor was noticed at the time of mobilization. The tremor was a postural and “no-no” tremor rather than an intention or resting tremor. The head tremor caused difficulty in eating and in other activities of daily living. No abnormal results were obtained by magnetic resonance imaging of the brain. The tremor was resistant to drugs, including anti-Parkinson drugs and benzodiazepines, and was therefore difficult to treat.

## Background

Spinocerebellar ataxia type 6 (SCA6) is a type of spinocerebellar degeneration (SCD) characterized by autosomal dominant, adult-onset, slowly progressive cerebellar ataxia. SCA6 is caused by a CAG expansion in the *CACNA1A* gene and generally manifests in the form of pure ataxia [1–3]. There have been reports on anesthetic considerations for patients with SCD, including SCA6, but it is still a controversial topic [4, 5]. We report a case of a patient with SCA6 who developed refractory head tremor after volatile anesthesia combined with epidural anesthesia.

## Case presentation

The case patient was a 64-year-old female with a height of 151 cm and a weight of 59 kg. When the patient was 60 years old, dysarthria occurred, and gait instability appeared a year later. A detailed examination was performed at another hospital and revealed a CAG repeat expansion. She was diagnosed with SCA6.

At the age of 64, she was found to have a pancreatic head mass and was diagnosed with stage III pancreatic cancer. She was referred to our hospital for surgery. Subtotal stomach-preserving pancreaticoduodenectomy was planned.

On admission, she presented with limb ataxia with lower predominance, mildly increased deep tendon reflexes, and dysphagia. However, she could walk alone and do housework. She had no involuntary movement at that time. Laboratory tests, electrocardiogram, chest X-ray, ultrasound cardiography, and pulmonary function test results were unremarkable.

The patient showed dysphagia and required early ambulation. General anesthesia with desflurane combined with epidural anesthesia was planned. On the day of surgery, an epidural tube was inserted from T9/10 in the operating room, and general anesthesia was induced with intravenous propofol (1 mg/kg), remifentanil (0.25 μg/kg/min), and rocuronium (0.7 mg/kg). After the patient was intubated without complication, anesthesia was maintained with 4% desflurane, remifentanil (0.1 μg/kg/min), and rocuronium (10 mg/h). As intraoperative analgesia, 0.25% ropivacaine (3–4 ml/h) was administered from an epidural catheter. In case of intraoperative hypotension, isotonic crystalloid fluids, colloid fluids, or vasopressor agents were used, and we maintained her mean pressure at over 60 mmHg. During surgery, no serious problems occurred. At the end of surgery, neuromuscular relaxant agents were antagonized with sugammadex (2 mg/kg), and she was extubated. She was able to communicate soon and was transferred to the intensive care unit (ICU). For postoperative analgesia, 0.2% ropivacaine combined with fentanyl (3 μg/ml) was administered at a rate of 4 ml/h from an epidural catheter.

The duration of the operation was 8 h and 52 min, and the duration of anesthesia was 10 h and 21 min. The bleeding volume was 1855 ml. A total of 4660 ml of crystalloid fluids and 2000 ml of colloid fluids were infused. Two units of red blood cells were transfused.

The patient was stable in the ICU, but experienced postoperative nausea and vomiting (PONV). Thus, until postoperative day (POD) 2, she was treated with intravenous metoclopramide (up to 20 mg/day). She was discharged to ward at POD 4. However, a head tremor was noticed on the same day. The tremor did not improve for a month, and she was referred to a neurologist. The type of tremor was a postural and “no-no” tremor, not an intention or resting tremor. The head tremor stopped when the head contacted the bed. The head tremor caused difficulty in eating and in other activities of daily living. After consultation, brain magnetic resonance imaging did not reveal an infarction area or degeneration in the basal ganglia, including the pallidum and subthalamic nucleus. The tremor was resistant to treatment, including treatment with anti-Parkinson drugs and benzodiazepines.

## Discussion

SCA6 is a subtype of SCD characterized by autosomal dominant, adult-onset, slowly progressive cerebellar ataxia, dysarthria, and nystagmus. Unlike other types of SCD, such as multiple system atrophy, SCA6 is a predominantly cerebellar disorder with less involvement of non-cerebellar systems [1, 3]. However, SCA6 has a wide spectrum, and a case with extrapyramidal symptoms, including parkinsonism and autonomic dysfunction, has been reported [2]. The mean age range of onset for SCA6 is 43–52 years, the prevalence of this disease is estimated to range from 0.02 to 0.31 per 100,000 people, and it has been reported to vary by geographic area [1].

Initial symptoms are gait unsteadiness, stumbling, imbalance, and dysarthria. Eventually, all patients develop gait ataxia, upper-limb incoordination, intention tremor, and dysarthria. Dysphagia is common. Hyperreflexia and extensor plantar responses occur in up to 40–50% of patients. Mentation is generally preserved [1].

A recent case study reported that there was no evidence of exacerbation of the pathological processes of SCD after neuraxial anesthesia [4]. Administration of epidural labor analgesia to a patient with olivopontocerebellar degeneration has been reported [6]. Similarly, labor analgesia with epidural anesthesia has been reported in spinocerebellar atrophy [7].

In this case, we considered the long duration of the operation and the necessity of early awakening after surgery. Because of a meta-analysis reporting that desflurane reduces operating room recovery time relative to propofol [8], we decided to manage the patient mainly with desflurane. However, there have been reports that halothane and enflurane each produce a reversible decrease in cerebellar cGMP and affect cerebellar mechanisms controlling motor activity in mice [9, 10]. Thus, some authors suggest that volatile agents should be administered carefully in patients with SCD [5].

To our knowledge, there are no reports of acute onset or exacerbation of symptoms after surgery or anesthesia in patients with SCD. However, there have been reports of patients with asymptomatic SCA10 developing cerebellar ataxia in pregnancy and in the postpartum period or after corticosteroid therapy [11, 12]. The authors of the previous reports hypothesized that hormonal changes are likely to influence the manifestation of the condition. On the other hand, factors related to stress might play a pivotal role in the development of acute cerebellar ataxia symptoms in patients with asymptomatic SCA10 [11].

Metoclopramide, which blocks the activity of the D2 dopamine receptor, was prescribed (up to 20 mg/day) for the treatment of PONV immediately after surgery until POD 2. However, the duration of metoclopramide therapy before the onset of extrapyramidal symptoms was short, and the dose of metoclopramide was low compared to the duration and doses previously reported, and furthermore, metoclopramide discontinuation could not resolve the head tremor [13, 14].

There is a possibility that the head tremor occurred soon after surgery, because the tremor stopped when the head contacted the bed. Delay of ambulation after surgery may complicate discovery of the head tremor in this case. Surgery or anesthesia might have led to the head tremor. First, certain anesthetic agents, such as volatile agents, may strongly influence the cerebellum or the extrapyramidal system in patients prone to developing neurodegenerative diseases. A previous report suggested that the function of p-type calcium channel alters in SCA6, which may contribute to degeneration of Purkinje cells [15]. Furthermore, a report have described that volatile agents may induce abnormal calcium release from the endoplasmic reticulum and neural cell damage, although desflurane has less potency compared to isoflurane [16]. Thus, there is a possibility that alterations in intracellular calcium homeostasis may have exacerbated her symptoms. Second, oxidative stress from surgery or anesthesia might be related. It was suggested that patients with SCA have mitochondrial dysfunction, and a previous study found elevated oxidative stress and significant mitochondrial alternations in the Purkinje cells in patients with SCA1 [17]. Third, unrecorded changes such as hypotension, anemia, or anorexia during or after surgery, may damage the brain in manners that are not apparent with imaging. Finally, the onset of the head tremor could have occurred by chance.

## Conclusions

We experienced a case of a patient with SCA6 who developed refractory head tremor after volatile anesthesia combined with epidural anesthesia. However, it is difficult to determine whether this case was an exacerbation of the existing disease or the onset of an independent extrapyramidal disease. For the safe anesthetic management of patients with SCA6, accumulation of further reports that include information on postoperative course is required.

## References

[1] Gomez CM, Adam MP, Ardinger HH, Pagon RA, Wallace SE, Bean LJH, Mefford HC, Stephens K, Amemiya A, Ledbetter N (2013). Spinocerebellar ataxia type 6. GeneReviews.

[2] Park H, Kim HJ, Jeon BS (2015). Parkinsonism in spinocerebellar ataxia. Biomed Res Int.

[3] Takeichi N (2014). Spinocerebellar degeneration (SCD). Equilibrium Res.

[4] Vadhanan P, Kumar P (2011). Anesthetic management of a patient with spinocerebellar degeneration. J Anaesthesiol Clin Pharmacol.

[5] Yokoshima Y, Habuka K, Omi S, Ohashi M, Watanabe Y (2005). Anesthetic management of a patient with spinocerebellar degeneration undergoing surgical repair of abdominal aortic aneurysm. J Clin Anesth.

[6] Tsen LC, Smith TJ, Camann WR (1997). Anesthetic management of a parturient with olivopontocerebellar degeneration. Anesth Analg.

[7] Rofaeel A, Balki M, Carvalho JC (2007). Carvalho: successful labour epidural analgesia in a patient with spinocerebellar ataxia. Can J Anesth.

[8] Wachtel RE, Dexter F, Epstein RH, Ledolter J (2011). Meta-analysis of desflurane and propofol average times and variability in times to extubation and following commands. Can J Anaesth.

[9] Triner L, Vulliemoz Y, Verosky M, Alpert M (1981). Halothane effect on cGMP and control of motor activity in mouse cerebellum. Anesthesiology.

[10] Vulliemoz Y, Verosky M, Alpert M, Triner L (1983). Effect of enflurane on cerebellar cGMP and on motor activity in the mouse. Br J Anaesth.

[11] Moro A, Munhoz RP, Raskin S, Bezerra TC, Moscovich M, Ashizawa T, Teive HA (2013). Acute onset of cerebellar ataxia in a spinocerebellar ataxia type 10 patient after use of steroids. Arq Neuropsiquiatr.

[12] Teive HA, Arruda WO, Raskin S, Munhoz RP, Zavala JA, Werneck LC, Ashizawa T (2011). Symptom onset of spinocerebellar ataxia type 10 in pregnancy and puerperium. J Clin Neurosci.

[13] Indo T, Ando K (1982). Metoclopramide-induced parkinsonism. Clinical characteristics of ten cases. Arch Neurol.

[14] Sethi KD, Patel B, Meador KJ (1989). Metoclopramide-induced parkinsonism. South Med J.

[15] Toru S, Murakosh T, Ishikawa K, Saegusa H, Fujigasaki H, Uchihara T, Nagayama S, Osanai M, Mizusawa H, Tanabe T (2000). Spinocerebellar ataxia type 6 mutation alters P-type calcium channel function. J Biol Chem.

[16] Yang H, Liang G, Hawkins BJ, Madesh M, Pierwola A, Wei H (2008). Inhalational anesthetics induce cell damage by disruption of intracellular calcium homeostasis with different potencies. Anesthesiology.

[17] Liu Z, Zhou T, Ziegler AC, Dimitrion P, Zuo L (2017). Oxidative stress in neurodegenerative diseases: from molecular mechanisms to clinical applications. Oxidative Med Cell Longev.

